# *In Vitro* Activity of Iclaprim against Methicillin-Resistant *Staphylococcus aureus* Nonsusceptible to Daptomycin, Linezolid, or Vancomycin: A Pilot Study

**DOI:** 10.1155/2017/3948626

**Published:** 2017-12-17

**Authors:** David B. Huang, Stephen Hawser, Curtis G. Gemmell, Daniel F. Sahm

**Affiliations:** ^1^Motif BioSciences, New York, NY, USA; ^2^Rutgers New Jersey Medical School, Trenton, NY, USA; ^3^IHMA Europe Sàrl, Route de I'Ile-au-Bois 1A, 1870 Monthey, Valais, Switzerland; ^4^Department of Bacteriology, University of Glasgow Medical School, Glasgow, UK; ^5^IHMA, Schaumburg, IL, USA

## Abstract

Iclaprim is a bacterial dihydrofolate reductase inhibitor in Phase 3 clinical development for the treatment of acute bacterial skin and skin structure infections and hospital-acquired bacterial pneumonia caused by Gram-positive bacteria. Daptomycin, linezolid, and vancomycin are commonly used antibiotics for these indications. With increased selective pressure to these antibiotics, outbreaks of bacterial resistance to these antibiotics have been reported. This *in vitro* pilot study evaluated the activity of iclaprim against methicillin-resistant *Staphylococcus aureus* (MRSA) isolates, which were also not susceptible to daptomycin, linezolid, or vancomycin. Iclaprim had an MIC ≤ 1 µg/ml to the majority of MRSA isolates that were nonsusceptible to daptomycin (5 of 7 (71.4%)), linezolid (26 of 26 (100%)), or vancomycin (19 of 28 (66.7%)). In the analysis of time-kill curves, iclaprim demonstrated ≥ 3 log_10_ reduction in CFU/mL at 4–8 hours for tested strains and isolates nonsusceptible to daptomycin, linezolid, or vancomycin. Together, these data support the use of iclaprim in serious infections caused by MRSA nonsusceptible to daptomycin, linezolid, or vancomycin.

## 1. Background

Iclaprim represents a diaminopyrimidine that inhibits bacterial dihydrofolate reductase of Gram-positive pathogens [1, 2]. Iclaprim exhibits potent *in vitro* activity against Gram-positive pathogens associated with acute bacterial skin and skin structure infections (ABSSSIs) and nosocomial pneumonia including *Staphylococcus aureus*, *Enterococcus* spp., and *Streptococcus* spp. [1]. Iclaprim demonstrates rapid *in vitro* bactericidal activity in time-kill studies in human plasma [3]. Iclaprim is in Phase 3 clinical development for the treatment of ABSSSI and nosocomial pneumonia. Daptomycin, linezolid, and vancomycin are commonly used antibiotics for these indications (daptomycin is indicated for ABSSSI but not indicated for nosocomial pneumonia); however, increased selective pressure to these antibiotics has resulted in outbreaks of bacterial resistance to these antibiotics. Because of this emerging resistance, this current study was done to evaluate iclaprim's activity against MRSA isolates that were nonsusceptible to daptomycin, linezolid, or vancomycin.

## 2. Materials and Methods

Antibacterial susceptibility testing was conducted at the Department of Bacteriology, Glasgow Royal Infirmary, Glasgow, Scotland [4], and Eurofins Microbiology Laboratories on a range of MSSA and MRSA strains and isolates with varying susceptibilities to several recognized antistaphylococcal antibiotics. A total of 61 nonduplicative, nonconsecutive isolates of methicillin-resistant *S. aureus* (MRSA), which were nonsusceptible to daptomycin, linezolid, or vancomycin, were obtained from Eurofins repository or from the National Institutes of Health Network on Antimicrobial Resistance to *Staphylococcus aureus* (NARSA) repository.

Clinical isolates were identified by the submitting laboratories, and identifications were confirmed centrally at Eurofins using the Bruker matrix-assisted laser desorption ionization-time of flight mass spectrometry (MALDI-TOF) biotyper. Susceptibility testing and minimal inhibitory concentration (MIC) interpretations were performed according to broth microdilution protocols. *S. aureus* breakpoints for daptomycin, linezolid, and vancomycin are ≤1, ≤4, and ≤2 µg/mL (4–8 µg/mL were classified as vancomycin-intermediate *S. aureus* and ≥16 were classified as vancomycin-resistant *S. aureus*), respectively. To date, there are no published clinical breakpoints for iclaprim. However, based on a number of factors (e.g., MRSA distribution of MICs, assessment of the pharmacokinetics/pharmacodynamics of iclaprim, and the study of the clinical outcomes of MRSA infections when iclaprim was used in Phase 2 and 3 studies) outlined in the CLSI M23 guideline, an iclaprim MIC ≤ 1 µg/mL for *S. aureus*, including MRSA, has been proposed.

MRSA isolates were tested in cation-adjusted Mueller–Hinton broth (CA-MHB). Quality control and interpretation of results were performed in accordance with CLSI M100-S25 methods [5]. QC ranges for iclaprim were those approved by CLSI and published in M100-S25 [4]. Iclaprim and comparator antibiotic MIC results were within the CLSI published ranges against *S. aureus* ATCC 29213. Isolates were tested with MIC panels (Thermo Fisher Scientific, Cleveland, Ohio, USA) of comparator antibiotics (trimethoprim, trimethoprim-sulfamethoxazole, ceftriaxone, erythromycin, levofloxacin, oxacillin, meropenem, tetracycline, tigecycline, vancomycin, linezolid, and daptomycin).

The analysis of time-kill curves was performed by exposing 10^5^-10^6^ CFU/mL of each MRSA isolate or strain to iclaprim, daptomycin, linezolid, or vancomycin at 2, 4, and 8x MICs. Bactericidal activity was defined as a  ≥ 3 log_10_ reduction in CFU/mL after 24 hours of incubation.

## 3. Results

The MIC_50_ and MIC_90_ values for the MRSA nonsusceptible to daptomycin, nonsusceptible to linezolid, vancomycin-intermediate, and vancomycin-resistant were >1/>1, 8/>8, 4/8, and >32/>32 µg/mL, respectively. Among the seven MRSA isolates nonsusceptible to daptomycin (all seven had an MIC > 1 to daptomycin), four, two, and one had a vancomycin MIC of 4, 8, and 2 µg/mL, respectively.

**Figure 1:**
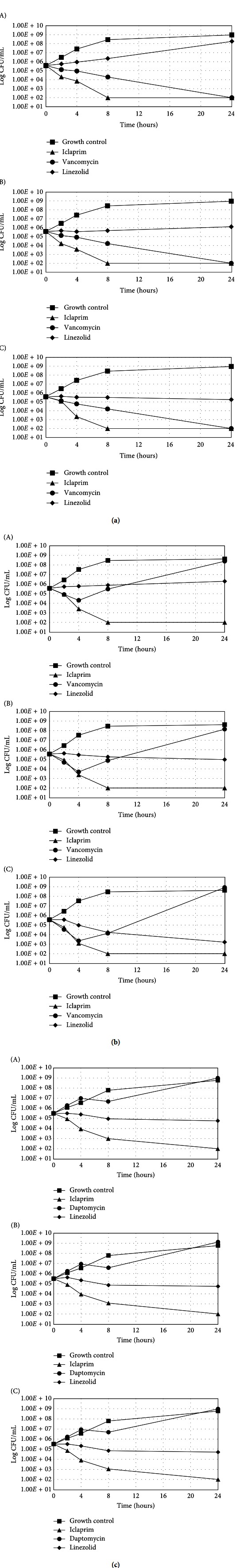
Iclaprim time-kill curves against MRSA isolates nonsusceptible to linezolid, resistant to vancomycin, and nonsusceptible to daptomycin, 2x, 4x, and 8x MICs were used for all antibiotics. (a) MRSA, linezolid nonsusceptible strain (MIC ≥ 8 µg/mL), ATCC 986537, NRS271. (A) 2x MIC. (B) 4x MIC. (C) 8x MIC. Iclaprim showed significantly lower CFU at 2 h, 4 h, 8 h, and 24 h compared to control, vancomycin, and linezolid (*P* < 0.01; one-way ANOVA with Tukey's post hoc test). (b) MRSA, vancomycin-resistant strain (MIC ≥ 32 µg/mL), ATCC 1409053, vanA positive. (A) 2x MIC. (B) 4x MIC. (C) 8x MIC. Iclaprim showed significantly lower CFU at 4 h, 8 h, and 24 h compared to control, vancomycin, and linezolid (*P* < 0.01; one-way ANOVA with Tukey's post hoc test). (c) MRSA, daptomycin-resistant strain (MIC ≥ 4 µg/mL) (clinical isolate). (A) 2x MICs. (B) 4x MIC. (C) 8x MIC. Iclaprim showed significantly lower CFU at 4 h, 8 h, and 24 h compared to control, daptomycin, and linezolid (*P* < 0.01; one-way ANOVA with Tukey's post hoc test).

Table 1 shows that iclaprim exhibited potent activity against the majority of the 61 MRSA isolates that were nonsusceptible to daptomycin, linezolid, or vancomycin (MIC_50_ 0.25 µg/mL). In the Glasgow study, all strains and isolates of MRSA and MSSA had an iclaprim MIC ≤ 1 µg/mL. Iclaprim notably exhibited 100% activity against MRSA isolates (*n* = 26) that were nonsusceptible to linezolid. A total of 9 (15.2%) isolates had reduced susceptibility to iclaprim with MICs > 8 µg/mL (Table 1). These isolates were not clustered in time of isolate collection, infection type, and/or geographic region.  Figure 1 shows representative time-kill curves of iclaprim (2x, 4x, and 8x MICs for all antibiotics), which exhibited bactericidal activity at 4–8 hours against MRSA strains and isolates nonsusceptible to daptomycin, linezolid, or vancomycin. As expected, representative time-kill curves of daptomycin exhibited no activity against MRSA strains and isolates nonsusceptible to daptomycin, linezolid exhibited no activity against MRSA strains and isolates nonsusceptible to linezolid, and vancomycin exhibited no activity against MRSA strains and isolates nonsusceptible to vancomycin.

## 4. Discussion

This report shows that iclaprim, without a synergistic combination of a sulfonamide, was highly active and rapidly bactericidal against a collection of 61 MRSA clinical isolates with nonsusceptible phenotypes to daptomycin, linezolid, or vancomycin. The MIC_50_ value of 0.25 µg/mL for MRSA documented in this study was consistent with MIC_50_ values in two previous surveillance reports for 5937 Gram-positive isolates, including MRSA, beta-hemolytic streptococci (most commonly *Streptococcus pyogenes* and *S. agalactiae*), and *S. pneumoniae* [1]. These isolates were collected from patients in the US and EU with skin and soft tissue, blood stream, and respiratory clinical specimens.

Based on MIC distributions of MRSA, assessment of the pharmacokinetics and pharmacodynamics of iclaprim, and the study of the clinical outcomes of MRSA infections when iclaprim was used in Phase 2 and 3 studies, an iclaprim MIC ≤ 1 µg/mL for *S. aureus*, including MRSA, has been proposed as the breakpoint for nonsusceptibility. The 80 mg fixed dose is based on prior animal models of infection studies, which suggest that the pharmacokinetic and pharmacodynamics (PK/PD) drivers, which best correlated with efficacy, were the area under the curve from 0 to 24 hours at steady state (AUC_0–24 h,ss_), AUC/minimum inhibitory concentration (MIC), and time above the MIC during the dosing interval (T > MIC). In addition, using PK data collected from 470 patients from a Phase 3-complicated skin and skin infection (cSSSI) trials (ASSIST-1 and 2), population PK modeling, and Monte Carlo simulation identified that the fixed iclaprim 80 mg dosage regimen optimally maximized AUC_0–24 h,ss_, AUC/MIC, and T > MIC while minimizing the probability of a *C*_max,ss_ to ≥ 800 ng/mL, a concentration associated with dose-limiting toxicity [6]. Based on PK/PD analyses, iclaprim 80 mg administered over two hours every 12 hours adequately covers *S. aureus* clinical isolates with an iclaprim MIC ≤ 1 µg/mL; therefore, this dose was selected as the dosing scheme for ongoing Phase 3 clinical trials.

A limitation of this study is the small numbers of daptomycin and linezolid nonsusceptible and vancomycin-resistant MRSA strains to arrive at conclusive activity of iclaprim against these types of strains and dose selection justification for clinical trials, which robust *in vitro* data are necessary. However, these data suggest that larger studies are warranted in examining iclaprim's activity against daptomycin and linezolid nonsusceptible and vancomycin-resistant MRSA. The findings of reduced daptomycin susceptibility and reduced vancomycin susceptibility and resistance have been reported in *S. aureus*. Daptomycin and vancomycin cross-resistance is believed to be related to the physical barrier of a thickened cell wall of MRSA against the penetration of daptomycin and vancomycin molecules [7, 8]. A possible reason as to why iclaprim had reduced activity against such isolates may relate to the mechanism of action of iclaprim, which interferes with folate metabolism in the bacterial cell by competitively blocking the biosynthesis of tetrahydrofolate. This product acts as a carrier of one-carbon fragments and is necessary for the ultimate synthesis of DNA, RNA, and bacterial cell wall proteins. As vancomycin-resistant strains are already altered in terms of cell wall targets, it is likely that some products of folate metabolism are less important [9].

The results of this *in vitro* study suggest that iclaprim may be a useful treatment option for infections caused by MRSA, including those with nonsusceptible phenotypes to daptomycin, linezolid, or vancomycin. Daptomycin, linezolid, and vancomycin are antibiotics that are FDA approved, and the Infectious Diseases Society of America guidelines list these antibiotics as treatment options for skin and skin structure infections (SSSIs) caused by Gram-positive pathogens [10]. New therapeutic options are needed, especially because of reported nonsusceptibility of Gram-positive bacteria to daptomycin, linezolid, and vancomycin and its associated poor outcomes, increased length of stay, healthcare costs, and overall morbidity [11–14].

## 5. Conclusion

In conclusion, the results from this pilot *in vitro* study show the potent and rapid bactericidal activity of iclaprim against clinical MRSA isolates, including those with nonsusceptible phenotypes to daptomycin, linezolid, or vancomycin. Continued surveillance is warranted to track the continued potency of iclaprim, as well as MRSA isolates nonsusceptible to daptomycin, linezolid, and vancomycin and to detect any potential emergence of resistance.

## Figures and Tables

**Table 1:** Iclaprim *in vitro* activity against MRSA isolates nonsusceptible to daptomycin, linezolid, or vancomycin.

MRSA phenotype (number)	Iclaprim MIC ≤ 1, µg/mL (%)	Iclaprim MIC_50_/MIC_90_ (µg/mL)	MIC range (µg/ml)
Daptomycin nonsusceptible (*n* = 7)	5/7 (71.4)	0.25/>8	0.12–>8
Linezolid nonsusceptible (*n* = 26)	26/26 (100.0)	0.06/0.25	0.03–1
Vancomycin intermediate (*n* = 23)	16/23 (69.6)	0.25/>8	0.25–>8
Vancomycin resistant (*n* = 5)	3/5 (60.0)	0.5/>8	0.25–>8
